# The complete mitochondrial genome of *Hemitripterus villosus* (Pallas, 1814) from Zhoushan archipelago

**DOI:** 10.1080/23802359.2021.1962763

**Published:** 2021-08-11

**Authors:** Keyue Shen, Hao Zhang, Shiyan Feng, Chenchen Wang, Shengyong Xu

**Affiliations:** Fisheries College, Zhejiang Ocean University, Zhoushan, China

**Keywords:** Hemitripteridae, mitogenome, *Hemitripterus villosus*, whole genome sequencing

## Abstract

In this study, we used whole genome sequencing to obtain the complete mitochondrial genome of *Hemitripterus villosus*. This mitochondrial genome, consisting of 17,449 base pairs (bp), contains 13 protein-coding genes, 2 ribosomal RNAs, 22 transfer RNAs and 2 noncoding control regions (control region and origin of light-strand replication) as those found in other vertebrates. Control region, of 1799 bp in length, is located between tRNA^Pro^ and tRNA^Phe^. We identified short tandem repeat sequences in the control region, which contributed largely to the relatively long mitogenome. The complete mitogenome data provides useful genetic markers for the studies on the molecular identification, phylogenetic analysis and conservation genetics.

The sea raven *Hemitripterus villosus* (Pallas, 1814) (NCBI:txid255551), which belongs to order Perciformes, suborder Cottioidei and family Agonidae, is one of the representative species in the Northwest Pacific, especially in coastal waters of China, Korea, and Japan (Jang et al. 2019; Wang et al. 2020). In addition, this species is an important commercial species in Chinese and Korea fishery industries (Jang et al. 2019). However, despite its ecological and economical importance, sparse genetic and genomic data of sea raven have been reported till now. Complete mitochondrial genomes are valuable molecular markers for phylogenetics and population genetics studies. To facilitate the phylogenetics and genetic variation studies, we present the complete mitogenome sequence of *H. villosus* in the present study.

The sample of *H. villosus* was collected from the coastal water of Zhoushan (30.0772°N, 122.3785°E), China in March 2020. The examined specimen was preserved at Fisheries Ecology and Biodiversity Laboratory and the marine life herbarium in Zhejiang Ocean University (Dr. Shengyong Xu, kevin890223@163.com) under specimen accession Nos. ZJOU-07547 and ZJOU-MLH-575412, respectively. The genomic DNA was extracted from dorsal-lateral muscles (30 mg) using Rapid Animal Genomic DNA Isolation Kit (Sangon Biotech Co., Ltd., Shanghai, CN). A genomic library was established and followed by next-generation sequencing. Whole genome sequencing (sequencing depth 50X) was conducted by using Illumina Hiseq4000 platform with the sequencing insertion of 350-bp. The whole genome sequencing data were deposited in the short read archive (SRA) database under accession number PRJNA746401. Quality check for sequencing data was done by FastQC (Andrews 2010) and the filtered clean data were assembled and mapped to complete mitogenome sequence using NOVOPlasty v3.7.2 (Dierckxsens et al. 2017). Subsequently, the assembled sequence was annotated using the online Mitochondrial Genome Database of Fish server (Iwasaki et al. 2013) and the MITOS Web Server (Bernt et al. 2013).

The final sequence has been deposited in GenBank with accession number MW836107. The complete mitochondrial genome of *H. villosus* (17,449 bp in length) consists of 13 protein-coding genes, 22 tRNA genes, 2 rRNA genes and 2 non-coding control regions (control region and origin of light-strand replication). The arrangement of all genes is identical to those of most teleosts (e.g. Chen 2013; Chiang et al. 2013; Wang et al. 2008). Most of the genes are encoded on the heavy strand (H-strand), except for the eight tRNA genes (i.e. -Gln, -Ala, -Asn, -Cys,-Tyr, -Ser(UGA), -Glu and -Pro) and one protein-coding gene (ND6). The overall base composition is 26.16% of T, 29.47% of C, 27.33% of A and 17.03% of G, with a slight A + T-rich feature (53.48%). Except for COI starting with GTG, the remaining 12 protein-coding genes start with ATG. It is important to note that some of the protein-coding genes (7 of 13 genes) are inferred to terminate with an incomplete stop codon (COII, COIII, ND2, ND3, ND4, ATPase 6 and Cyt *b*), with six (COI, ATPase8, ND1, ND4L, ND5 and ND6) sharing TAA as a stop codon, respectively. These features are common among vertebrate mitochondrial genome, and TAA is supposed to be appeared via posttranscriptional polyadenylation (Ojala et al. 1981). The two ribosomal RNA genes, 12S rRNA (951 bp) and 16S rRNA (1695 bp), are located between tRNA^Phe^ and tRNA^Leu^. The non-coding control region (D-loop) is 1799 bp in length, and is located between tRNA^Pro^ and tRNA^Phe^. Within D-loop, a termination-associated sequence (TAS), conserved sequence blocks (CSB-1, CSB-2 and CSB-3), and several areas of highly conserved sequence (CSB-F, CSB-E and CSB-D) were detected. Besides, we also identified short tandem repeat sequences (STRs) in the first hypervariable region of control region. A total of 5 repeats were identified and the repeat unit was ‘TTT CTT AGA CAA TTA TTA AAC ACT TTA AAA CCA CTT TAA AAA CAT TTT AAA AAC ATT TTA AAA ACA TTT TTT AAA ACG TCT TAC TAA ATA ATA’. The tandem repeat sequences in control region should contribute largely to the relatively long mitogenome of the investigated individual. Given the short read length of Illumina sequencing, the accuracy of identified STR was further confirmed by using Sanger sequencing with the designed primers (forward primer: ACTCATCAAACACCCATCA; reverse primer: ATACTCCTACTAAGTTAC). Also, further studies are needed to confirm the heterogeneity of STRs among individuals collected from different geographic populations.

Phylogenetic relationships were constructed using Neighbor-joining (NJ) algorithm for 1000 replicates in MEGA 6 (Tamura et al. 2013) among 10 species of suborder Cottioidei based on 13 mitochondrial protein-coding genes (Figure 1). By aligning amino acid sequences, our reconstructed phylogenetic topology showed that *H. villosus* has a relatively close relationship with *Blepsias cirrhosis*, which also belongs to family Agonidae. Besides, our results revealed a clear phylogeny of these families in suborder Cottioidei: (Agonidae, (Hexagrammidae, Cottidae)). The information of the mitogenome will be useful for future phylogenetic studies and specimen identification of species in order Perciformes.

**Figure 1.**
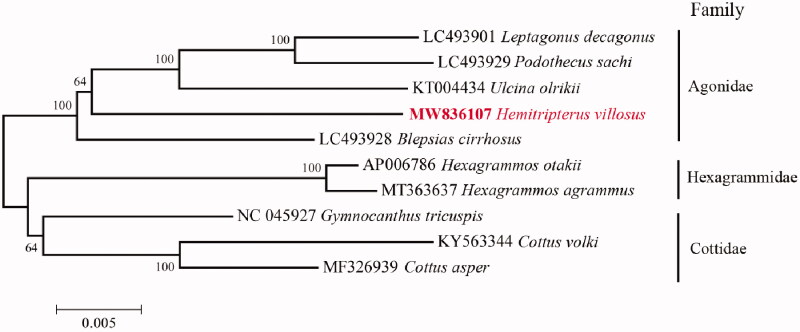
Neighbor-joining (NJ) topology for 10 species of suborder Cottioidei based on 13 mitochondrial protein-coding genes.

## Data Availability

The genome sequence data that support the findings of this study are openly available in GenBank of NCBI at [https://www.ncbi.nlm.nih.gov] (https://www.ncbi.nlm.nih.gov/) under the accession no. MW836107. The associated BioProject, SRA, and Bio-Sample numbers are PRJNA746401, SRR15129992, and SAMN20205129 respectively.
